# Tongguan Capsule Protects against Myocardial Ischemia and Reperfusion Injury in Mice

**DOI:** 10.1155/2013/159237

**Published:** 2013-09-01

**Authors:** Jianyong Qi, Juan Yu, Lei Wang, Liheng Guo, Shiyu Ma, Donghui Huang, Miao Zhou, Jiashin Wu, Minzhou Zhang

**Affiliations:** ^1^Intensive Care Laboratory, Guangdong Province Hospital of Chinese Medicine, 2nd Affiliated Hospital of Guangzhou University of Chinese Medicine, Guangzhou 510120, China; ^2^Animal Laboratory, Guangdong Province Hospital of Chinese Medicine, 2nd Affiliated Hospital of Guangzhou University of Chinese Medicine, Guangzhou 510006, China; ^3^Department of Oral and Maxillary Surgery, Stomatology Hospital of Guangzhou Medical University, Guangzhou 510140, China; ^4^University of South Florida, Tampa, FL 33612, USA

## Abstract

Myocardial ischemia/reperfusion (I/R) can induce lethal ventricular arrhythmia and myocardial infarction. One of the clinical strategies for managing patients with high risk of myocardial I/R is to prevent the occurrence of arrhythmias and limit the size of infarction following a coronary episode. Tongguan Capsule (TGC) is one of the popular herbal remedies in treating coronary artery disease in the clinics of Chinese medicine. However, the potential roles and mechanisms of TGC in reducing I/R injury are still unclear. The present study statistically assessed the effectiveness of TGC in reducing I/R injury by comparing the infarct size (IS), risk region (RR), and arrhythmia (in electrocardiogram) among four groups of surgically created mice models of myocardial I/R: SHAM, I/R, VER (I/R with verapamil 20 mg/kg pretreatment), and TGC (I/R with TGC 5 g/kg/d pretreatment). We found that IS was significantly smaller in the TGC and VER groups than I/R group, and the incidence of arrhythmias was reduced in the TGC group compared with I/R group, although there were no differences in RR among the four groups. We conclude that TGC is effective in reducing I/R injury in mice. These results provided an experimental basis for clinical application of TGC in reducing I/R injury.

## 1. Introduction

Acute myocardial infarction is the most common cause of cardiac death. Early reperfusion after coronary obstruction represents the most effective means of therapy. However, myocardial ischemia reperfusion (I/R) can induce lethal ventricular arrhythmia and myocardial infarction [1–3]. Nearly 50% of myocardial infarction occur following I/R [4]. In the United States alone, approximately 1 million people suffer from myocardial infarction every year. Additionally, 700 patients undergo cardioplegic arrest for various cardiac surgeries [5]. Despite the different etiologies that lead to partial or complete arrest of cardiac circulation, both patient groups share myocardial ischemia and reperfusion injury as a common pathophysiological feature [6]. Myocardial ischemia and reperfusion injury was first described by Jennings et al. in 1960 [7]. They observed that reperfusion accelerated the development of necrosis in a canine coronary ligation model with histological changes after 30 to 60 minutes of I/R comparable to the degree of necrosis normally seen after 24 hours of permanent coronary occlusion.

A traditional Chinese medicine, Tongguan Capsule (TGC), formulated mainly with the following herbs: *Astragalus mongholicus* and *salvia miltiorrhiza*, etc. [8, 9], has been widely used in China to treat patients with angina pectoris and acute coronary syndrome [10, 11]. A recent clinical trial [12] confirmed its effectiveness to improve symptoms and reduce angina pectoris and complications along with few adverse effects. Clinical trials demonstrated that TGC is effective and safe for treating angina pectoris [13]. TGC was shown to activate eNOS signaling pathway in a pharmacodynamic study [14].

Although effective for treating angina pectoris, the effects of TCG on myocardial I/R injury remain unclear. Our previous studies found that pretreatment with TGC at high doses for 3 days before pituitrin intraperitoneal injection (ip) reduced myocardial ischemia in rats [14]. The current study evaluated a hypothesis that TGC can protect heart against I/R injury. To evaluate this hypothesis, we examined the myocardial injury after ischemia and reperfusion in mouse hearts pretreated with TGC and compared the effects of TGC with control groups. We observed that TGC could significantly reduce the infarct size (IS) of I/R. Therefore, this study experimentally demonstrated that TGC is an effective Chinese herbal medicine to control and reduce I/R injury.

## 2. Materials and Methods

### 2.1. Animals and Reagents

This study was performed in accordance with the guidelines and with approval from the Institutional Animal Care and Use Committee of Guangdong Province Hospital of Chinese Medicine, Guangzhou University of Traditional Chinese Medicine, and with the Guide for the Care and Use of Laboratory Animals published by the National Academy of Sciences (8th edition, Washington DC, 2011).

Ten to twelve weeks old male wild-type C57BL/6J mice (25 ± 5 g body weight) were obtained from the Experimental Animal Center of Guangdong Province. Triphenyltetrazolium chloride (TTC) and Evans blue were purchased from DingGuo Biotechnology Corp (Beijing, China); 10% Neutral Buffered Formalin were purchased from WEX Corp (Guangzhou, China); Pentobarbital sodium were purchased from Sigma-Aldrich Corp. (Guangzhou, China); TGC was produced by Guangdong Province Hospital of Chinese Medicine (Guangzhou, China), batch number 100519.

### 2.2. I/R Model In Vivo

**Figure 1:**
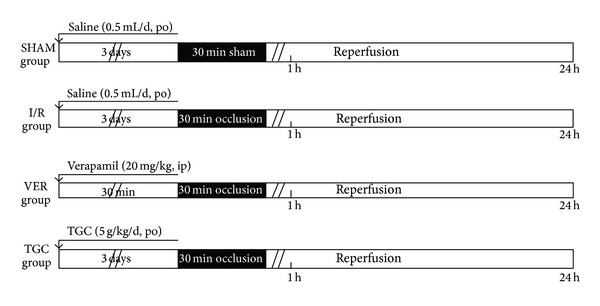
Experimental protocols. Four groups (SHAM, I/R, VER, and TGC groups) of mice were studied for infarct size analysis. Days 1–3: mice were subjected to either saline (SHAM and I/R groups) or TGC via direct gastric gavage daily. Day 4: mice in SHAM, I/R, TGC, and VER groups (verapamil 20 mg/kg i.p pretreatment) were subjected to a 30 min LAD occlusion. Day 5: after 24 h of reperfusion following LAD occlusion, all animals were sacrificed for subsequent measurement of infarct size.

The murine model of I/R has been previously described in detail [15, 16]. Briefly, mice were anesthetized with sodium pentobarbital (60 mg/kg, i.p.), intubated, and ventilated with room air at a rate of 110 strokes/min and with a tidal volume of 0.25 mL using a mouse ventilator (Inspira, Harvard Apparatus, Holliston, MS, USA). The chest was opened through a left thoracotomy with the aid of a dissecting microscope. An 8-0 nylon suture was passed under the mid-left anterior descending coronary artery (LAD, 2-3 mm from the tip of the left auricle), and a nontraumatic occluder was applied on the artery. Ischemia was elicited by a 30 min coronary occlusion followed by 24 hours reperfusion (Figure 1). Significant changes, including widening of the QRS complex and elevation of ST segment in electrocardiography, were indicators of successful coronary occlusion. The chest was closed in layers, and animals were weaned from the ventilator when they resumed spontaneous breathing [17, 18].

### 2.3. In Vitro Tissue Staining

At the end of 24-hour reperfusion, the heart was perfused with 1X phosphate buffer solution (1X PBS, pH 7.4) through an aortic cannula. The ligature around the LAD was retied. Two mL of 1% Evans blue dye was injected into the left coronary artery by reversing perfusion through the aorta, and the dye was circulated and uniformly distributed, except in the portion of the heart previously perfused by the occluded coronary artery (risk region, RR). The heart was quickly excised, and both atria and the right ventricle were removed. The left ventricle was weighed and sliced horizontally to yield six slices. After being weighted individually, the slices were incubated in 1% TTC prepared with 1X PBS for 8–15 minutes at 37°C, fixed in 10% neutral buffered formaldehyde for 24–48 h, and then photographed under a microscope with a digital camera [19].

### 2.4. Infarct Size (IS) Measurement

The areas stained with Evans blue (blue area, Normal Zone, NZ), TTC (red staining, Risk Region, RR), and TTC-negative area (white area, Infarct Size, IS) were measured digitally using Image Pro-plus (Version 6.0). The myocardial infarct size was measured and expressed as a percentage of infarct size over the total RR. We identified infarct, at-risk, and nonischemic areas based on tissue staining and measured infarct sizes by computerized video planimetry [20].

### 2.5. Heart Rhythm Analysis

Continuous electrocardiographic monitoring (RM6240; ChengDu Instruments) was performed during in vivo myocardial I/R with LAD ligation. Heart rate and rhythm were analyzed throughout the experiment. The incidence and type of arrhythmias, including atrial premature beats, heart block, and ventricular tachycardia, were evaluated during I/R based on limb lead recordings.

### 2.6. Statistical Analysis

Data are reported as mean ± SEM. Bonferroni's post hoc method was used to assess the significance of differences using GraphPad Prism version 4.0. Incidence of arrhythmia was evaluated by *χ* ^2^ test. A *P* value of <0.05 was considered statistically significant [21, 22].

## 3. Results

### 3.1. Protocol

To evaluate the cardioprotective effects of TGC on I/R injury, we firstly created a murine ischemia model as described earlier. Our previous study showed that the best protective effects to myocardial ischemia occurred when TGC was administrated 3 days before pituitrin intraperitoneal injection (ip) in rats [14]. Based on our previous study, literature, and clinical usage in patients (with dose conversion between humans oral usage and animals), TGC powder (Guangdong Province Hospital, Guangzhou, batch number 100519) at a dose of 5 g/kg body weight mixed with 0.5 mL saline was administered daily via direct gastric gavage, for 3 days prior to surgery. Four groups of mice were studied: SHAM group, I/R group, VER group and TGC group (Figure 1). Mice in I/R were subjected to 30 min of coronary occlusion followed by 24 h of reperfusion. VER group (verapamil group, 20 mg/kg, i.p) served as a positive control of the protective effects of drug ischemia preconditioning. The I/R group and SHAM group received saline (0.5 mL/day) for the same duration (3 days). To evaluate whether the high dose was safe for mice, we carried out a preliminary experiment by gavaging 2 mice for 3 days. Both mice survived and were healthy, so we proceeded with the current experiment.

### 3.2. Exclusions

A total of 47 mice were used in the current experiments. Three mice died (Table 1, 6% total mortality). This mortality was quite low for open-chest LAD ligation surgeries in mice. In all, twelve mice (26%) were excluded because of death (3 mice), severe bleeding during surgery (3 mice), technical problems (1 mouse, malfunction of the ventilation system, damage to the coronary vessels), or inadequate postmortem staining (4 mice). Thirty-five mice (74%) successfully completed the entire protocol and were included in the results (Table 1).

### 3.3. Risk Region and Infarct Size

**Figure 2:**
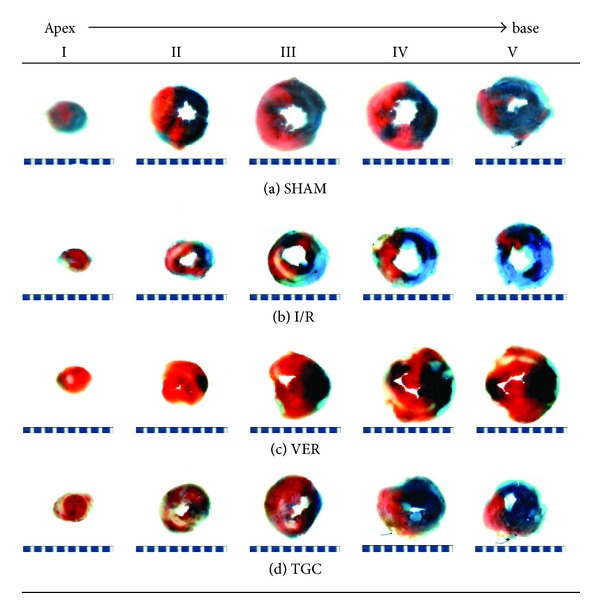
Example dye staining of the normal, risk, and infracted regions. Photomicrographs (×10) of heart sections obtained from mice subjected to myocardial ischemia/reperfusion (30 minutes/24 hours) treated with SHAM ((a), blank control), I/R ((b), negative control), VER ((c), positive control), and TGC (d). Blue-stained portion: nonischemic, normal region; red-stained portion: ischemic/reperfused, risk but not infarcted region; unstained portion (white area): ischemic/reperfused, infarcted region. Scale at bottom is in mm.

**Figure 3:**
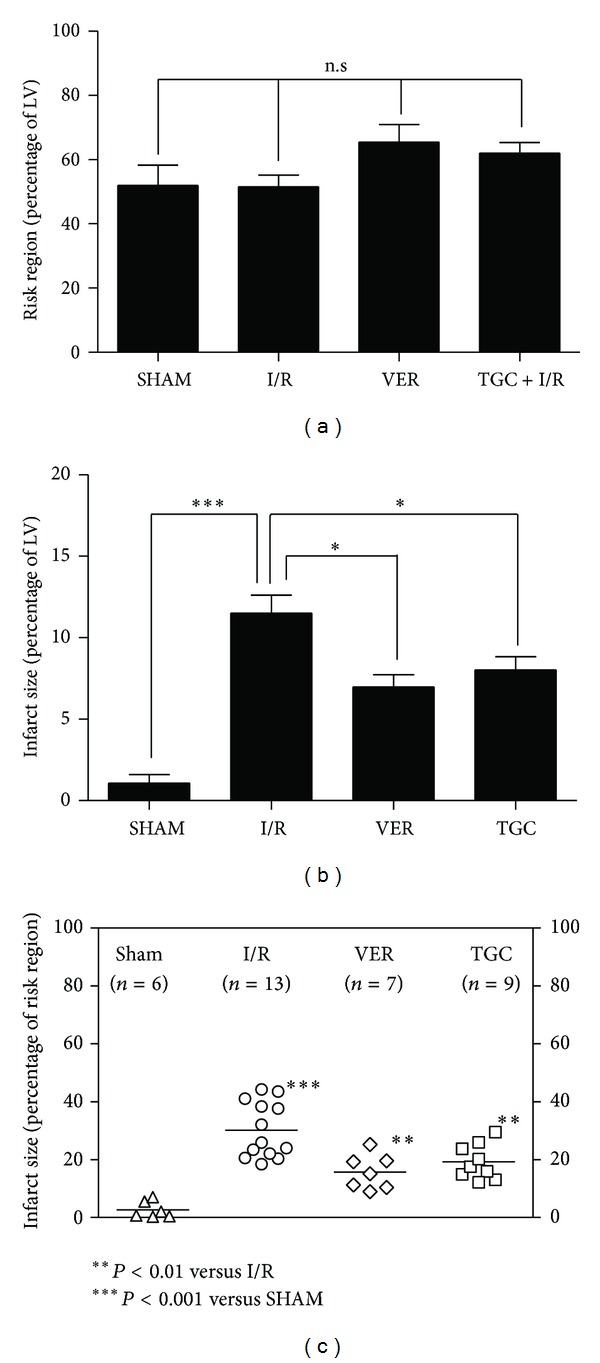
The risk regions and infarct sizes. (a) Myocardial risk region (RR) expressed as percent of left ventricle. (b, c) myocardial infarct sizes (IS) expressed as percent of total LV (b) and ischemic reperfused area ((c), risk region, RR). Data are presented as mean ± SEM, ***P < 0.001 I/R group compared with SHAM, *P < 0.05, **P < 0.01 IS (percentage of LV and RR) in TGC and VER groups versus I/R group, respectively.

We used infarct size, which is the gold standard for I/R injury evaluation, to investigate the cardioprotective effects of TGC on I/R injury. As shown in Figure 2, we can clearly differentiate infarct size (IS, white area) from risk region (RR, red area) and RR from normal zone (NZ, dark blue). As illustrated in Figure 3(a), there were no significant differences among the four groups in their LV weights and in their weights of the region at risk (Table 2). In the I/R group, the 30 min of coronary occlusion followed by 24 h of reperfusion resulted in an infarct size of 30.2 ± 2.7% of the region at risk (Figure 3(c)). In contrast, the TGC group had an infarct size of 19.3 ± 2.0% of the region at risk (7.8 ± 1.0% of LV) (Figure 3(b), Table 2). The VER group, which was the positive control group of protective effect of I/R injury, had an infarct size of 17.1 ± 2.4% of the RR (Figure 3(c)). Large, confluent areas of infarction spanned most of the thickness of the LV wall. Assessment of cell death at 24 h represented the final extent of myocardial infarction in this model (see the example of TGC group in Figure 2). The room temperature I/R surgery possibly helped in limiting the size of infarct. The small SEM (Figure 3(c)) and similar sizes of infarct in both groups indicated stability of this I/R model.

### 3.4. Arrhythmia

**Figure 4:**
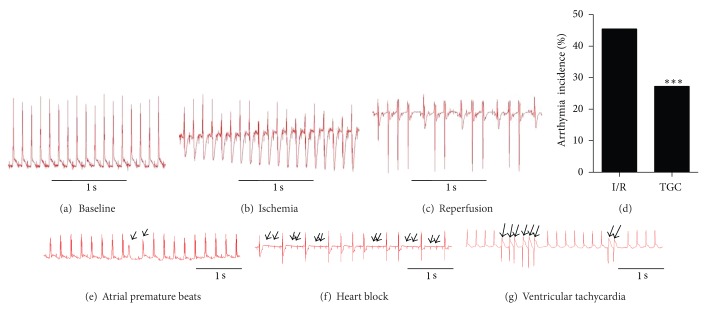
Reduced incidence of arrhythmia in TGC group. Representative ECG complexes in baseline (a), ischemia stage (b) and reperfusion stage (c). Ischemia produced dramatic alterations in the transmural ECG with a gradual increasing inversion of the T-wave and a decrease in the amplitude of R-wave (b). Premature ventricular contractions occurred frequently at the onset of reperfusion (c). Incidence of arrhythmia were reduced in TGC group (d), ***P < 0.01 versus I/R group (*χ* ^2^ test). Various types of arrhythmia, including atrial premature beats (e), heart block (f), and ventricular tachycardia (g) were observed during I/R injury.

Since I/R can induce both arrhythmia and myocardial infarction, we also analyzed the roles of TGC on occurrences of arrhythmia by examining the electrocardiogram (ECG) in the in vivo murine model of I/R injury. Figures 4(a)–4(c) illustrate three representative ECG complexes on the baseline, ischemia, and reperfusion stages. Ischemia dramatically altered transmural ECG. The most notable features were a gradual increasing inversion of the T-wave and a decrease in excitability as illustrated by the amplitude of R-wave (Figure 3(b)), subsequently followed by ST-segment elevation with continuous ischemia. Excitability recovered completely after 15 min of reperfusion. It is well recognized that reperfusion after ischemia is a powerful trigger of cardiac arrhythmias (with peak arrhythmia occurrences during reperfusion after 20–30 min ischemia) [2]. We observed that premature ventricular contractions occurred frequently at the onset of reperfusion in the I/R model (Figure 3(c)) in consistence with a previous report [23]. The results (Figure 4(d)) showed that I/R injury induced arrhythmias frequently in untreated hearts (incidence: 45.5%) but much less frequently in hearts pretreated with TGC (incidence: 27.2%). Thus, our results demonstrated that TGC was effective in reducing I/R-induced arrhythmias.

### 3.5. Correlations

**Figure 5:**
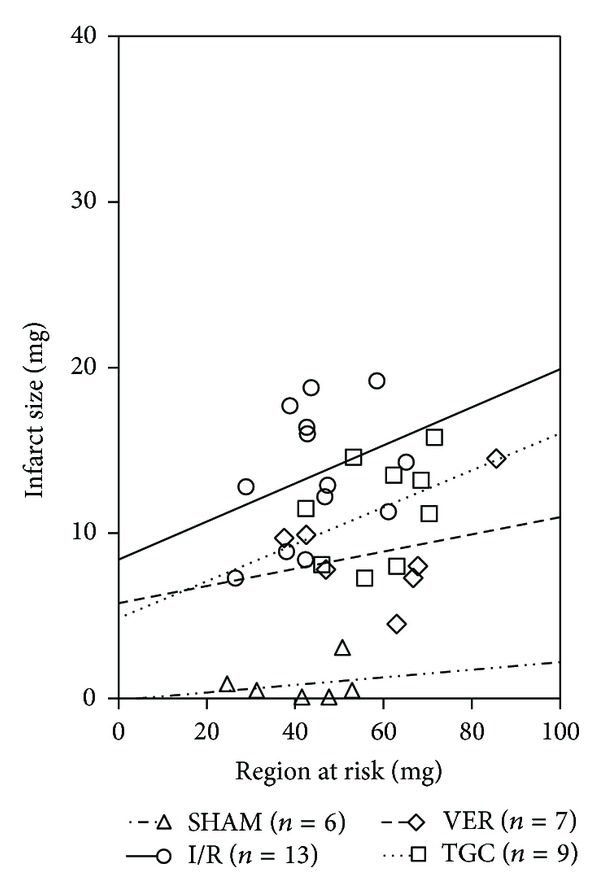
Relationships between the size of region at risk and the size of myocardial infarction. Linear regression analysis showed that the infarct size was unrelated to the size of the risk region in all groups. Linear regression equations: SHAM group, *y* = 0.02301*x* − 0.08, *r* = 0.23, *P* > 0.05; I/R group, *y* = 0.1151*x* + 8.4, *r* = 0.33, *P* > 0.05; VER group, *y* = 0.05201*x* + 5.767, *r* = 0.29, *P* > 0.05; TGC group, *y* = 0.1115*x* + 4.86, *r* = 0.38, *P* > 0.05.

To evaluate the relations between the risk region and infarct size, we performed linear analysis of their correlation. It is widely believed that bigger RR produces bigger IS [24]. However, our results showed that the size of the infarction was not linearly related to the size of the region at risk (*r* = 0.23, 0.33, 0.29, and 0.38 in the SHAM, I/R, VER, and TGC groups, resp.) (Figure 5). There was only a slight tendency of IS increase with RR. Considering similar RR in all I/R hearts, which was ~50% and independent from other variables, our data suggest that IS is a property of mice type and surgical interventions.

## 4. Discussion

This is the first study to investigate the cardioprotective effects of TGC against I/R injury in mice. We determined the effects of TGC on ischemic and reperfusion injury in an anaesthetized opened-chest murine model of acute myocardial ischemia. The results demonstrate that TGC markedly reduced the infarct size of reperfusion injury and the mortality of acute ischemia/reperfusion in mice.

The effects of against reperfusion injury by several cardioprotective drugs, including reactive oxygen species scavengers, calcium channel blockers, adenosine, and nicorandil, have recently been evaluated in clinical trials and found to be inadequate [25]. These unsatisfactory results could be due to the presence of multiple mechanisms of reperfusion injury and a lack of therapies targeting these mechanisms simultaneously. The complex profile of active ingredients in TGC may possibly overcome the deficiencies of these single-target drugs in protecting against I/R injury. One of our previous studies [14] reported that eNOS activity was increased in myocardium and serum after TGC administration. Astragaloside IV, one of the major components of TGC, was reported to protect against myocardial infarction by increasing the ATP-sensitive potassium current and by improving intracellular calcium handling [26–28]. Salvia miltiorrhiza (Danshen), another major component of TGC, has scavenging effects on free radicals and protects myocardial mitochondrial membrane from ischemia reperfusion injury [29]. Danshensu, which is an active component of Salvia miltiorrhiza, is considered effective in against myocardial ischemia/reperfusion injury and inhibits apoptosis of H9c2 cardiomyocytes via Akt and ERK1/2 phosphorylation [30]. Therefore, the protective effects of TGC against I/R injury can possibly be mediated through multiple signaling pathways.

We performed considerable amount of preliminary works before investigating the effects of TGC on MI/R injury. Because temperature can be a major determinant of infarct size [31, 32], this variable was evaluated in a preliminary study comparing mice with and without body temperature controlling (by using heating pads and heat lamps while continuously monitoring rectal temperature) throughout the experiment. We found 2 technical challenges that severely limited the use of body temperature controlling. The first one was time consuming to keep the mice at 36.4–37.6°C. The body temperature of mice usually decreased 2–4°C after anesthetizing and would fluctuate during the surgery. Secondly, using a heating pad and/or lamp to maintain a normal body temperature increased the rate of breathe and lowered the degree of anesthesia during the open-chest surgery and was associated with excessively high rate of postsurgery death. In our preliminary experiments, the death rate reached 80% when a heating device was used to keep normal body temperature during surgery. Because of this, we performed the surgery without a heating device, which reduced the death rate to 6% (see Table 1). Because all the four groups had the same protocol, thus, the effects of unheated body temperature during surgery would not affect the comparison among the groups.

The reliability of the measurements of infarct size was of paramount importance in the outcome of the present investigation. We implemented several modifications of the postmortem perfusion technique during the development of this protocol and achieved major improvements in the quality of tissue staining. The final protocol described in Figure 2 resulted in excellent staining and clear delineation of both region at risk and infarction, as demonstrated in Figure 2. Because of the quality of staining, the measurements of infarct size in this study were accurate and reproducible. The average infarct size in the I/R mice (negative control group) was 30.2 ± 2.7% of the region at risk in the current study, similar to the average infarct size of 34.4 ± 9.2% in I/R mice after the same I/R protocol (30 min coronary occlusion, 24 h reperfusion) reported by Michael et al. [33] and to the infarct size of 33.4 ± 4.5% in six I/R mice subjected to 30 min of occlusion and 2 h of reperfusion reported by Hutter et al. [34], although the infarct size is slightly smaller than observed by Guo et al. in mice [24] [50.9 ± 2.6% of the region at risk]. Differences in body temperature during surgery might contribute to the differences in the infarction sizes.

There were no statistical differences among our groups of mice in their heart-to-body weight ratios, age, body weight, and risk region (Table 2). A major concern in the design of these experiments was to ensure the results would be physiologically relevant. The minuscule size of the murine heart necessitates miniaturization of the procedures used in larger species and therefore poses a unique challenge in terms of maintaining general experimental conditions within normal values and avoiding artifacts.

## 5. Conclusion

The present study showed that a high loading dose of TGC 3 days before LAD ligation reduced the size of infarction by ischemia reperfusion injury. This result experimentally proved the effectiveness of TGC as a clinical therapy to protect against ischemia reperfusion injury at an early stage. Traditional Chinese medicine has been of great benefit to Asian people for centuries. However, evidence-based experimental verifications of their effectiveness and mechanistic studies have been insufficient. Experimental evidences of the TGC-induced cardioprotection can help to explain the improved outcomes after the immediate administration of TGC to patients with acute coronary syndrome and may lead to the development of better drugs and/or new therapeutic applications of TGC.

## Figures and Tables

**Table 1:** Reasons for excluding mice from study.

Group	SHAM	I/R	VER	TGC	Total
Bleeding	1	1	1	0	3
Death	0	1	2	1	4
Technical problems	0	1	0	0	1
Poor postmortem staining	0	2	1	1	4
Mice instrumented	6	13	7	9	35
Mice excluded	1	5	4	2	12
Mortality rate (%)	0	6	18	9	11
Mice included in study	6	13	7	9	35
Mice included in study (%)	86	72	64	82	74

SHAM group, I/R group, I/R + saline; VER group, I/R + verapamil, TGC group: I/R + TGC.

**Table 2:** Size of left ventricle, risk region and infarction in study.

	SHAM	I/R	VER	TGC
LV Wt, mg	99.0 ± 4.6	100.3 ± 4.9	90.7 ± 2.6	95.4 ± 2.9
Risk region Wt, mg	41.4 ± 4.6	44.8 ± 3.2	58.5 ± 6.4	59.2 ± 3.5
Infarct Wt, mg	0.9 ± 0.5	13.6 ± 1.1	9.8 ± 1.3	11.5 ± 1.0
Risk region, % of LV	51.9 ± 6.4	51.6 ± 3.6	65.5 ± 5.5	62.1 ± 3.2
Infarct, % of risk region	2.7 ± 1.2	30.2 ± 2.7^#^	17.1 ± 2.4**	19.3 ± 2.0**
Infarct, % of LV	1.1 ± 0.5	11.5 ± 1.1^#^	7.8 ± 1.0*	8.0 ± 0.8*

Data are mean ± SEM. LV Wt: left ventricular weight; RR: region at risk. There were no significant differences in the age, sex, and LV weight of the mice among the four groups. Also, there were no significant differences in the region at risk among the four groups. ^#^ *P* < 0.001 I/R group compared with SHAM, *P < 0.05, *P < 0.01 IS (percentage of LV and RR) in TGC and VER groups versus I/R group, respectively.
